# Effects of Antibodies in the Serum After the Administration of COVID Vaccines and Their Hematological and Cardiovascular Complications

**DOI:** 10.7759/cureus.47984

**Published:** 2023-10-30

**Authors:** Mehreen Haq, Sanjay V Deshpande

**Affiliations:** 1 Pathology, Jawaharlal Nehru Medical College, Datta Meghe Institute of Higher Education and Research, Wardha, IND; 2 Orthopaedics and Traumatology, Jawaharlal Nehru Medical College, Datta Meghe Institute of Higher Education and Research, Wardha, IND

**Keywords:** carditis, infection, titer, igg, anti-spike antibodies, vaccine, covid-19

## Abstract

The outbreak of COVID-19 was seen first in Wuhan, China, on December 31, 2019. Following this, the virus has emerged, mutated, and spread to all parts of the world, taking many lives. Scientists and healthcare workers all over the world have been involved in developing vaccines and drugs to prevent the deadly virus from spreading. In this review article, we focus on how the human body responds to immune responses by producing antibodies against our immune system and serum levels in different age groups. Few studies are being considered, which include data collected from adults in the UK community, health workers from Oxfordshire, studies from the UK, healthcare workers at a university healthcare center in Turkey, and lastly, non-seropositive and seronegative healthcare workers in the USA children's hospital, respectively, and their responses to the goal.

In addition to focusing on this study and its results, we also discuss the role of different vaccines and their development and antibody responses in the body due to natural and post-vaccine infections that include both doses in humans. We focus mainly on immunoglobulin M (IgM) and immunoglobulin G (IgG) levels in the serum produced by plasma cells, as they are involved in the first line of defense against the disease. With the development of effective vaccines and their production, trial, and market distribution to needy people, there are certain prospects for slowing down the progression of the virus, reducing mortality, and preventing re-infection in humans. However impactful and beneficial these vaccines have proven, they also carry a certain amount of danger to the people taking them. We also discuss in this article certain infrequent hematological and cardiovascular complications of the vaccine and their effect on the population.

## Introduction and background

Coronaviruses belong to a small family of viruses known as the *Coronavirinae*. They are well-thought-out, single-stranded ribonuclease acid (RNA) viruses with a round envelope, 100-160 nm wide, and a 27-32 kb gene. Of the seven different types of human coronaviruses, three are corona-causing viruses: the severe acute respiratory syndrome coronavirus (SARS-CoV), the Middle East respiratory syndrome coronavirus (MERs-CoV), and the novel coronavirus (SARS-CoV-2), known to cause severe infections involving the lung parenchyma and the respiratory system. Acute respiratory syndrome coronavirus 2 caused the deadly COVID-19. Initially named by the WHO in January 2020, COVID-19 has since sickened millions of individuals internationally, resulting in the deaths of a significant proportion of people [1,2]. Due to the virus's quick tendency to spread and the massive instances that have impacted the whole world's population, replicating the virus's proliferation has proven to be very difficult. Droplets from respiration and fomites are the main methods of viral transmission. Furthermore, virus propagation in the sewage was observed for up to five weeks following clinical recovery. Pyrexia and cough are the routinely acquired consequences of the virus. The average incubation period is four days. In addition to this, the patients have comorbidities such as high blood pressure, diabetes, and cardiac problems [3,4].

## Review

Methodology

**Figure 1:**
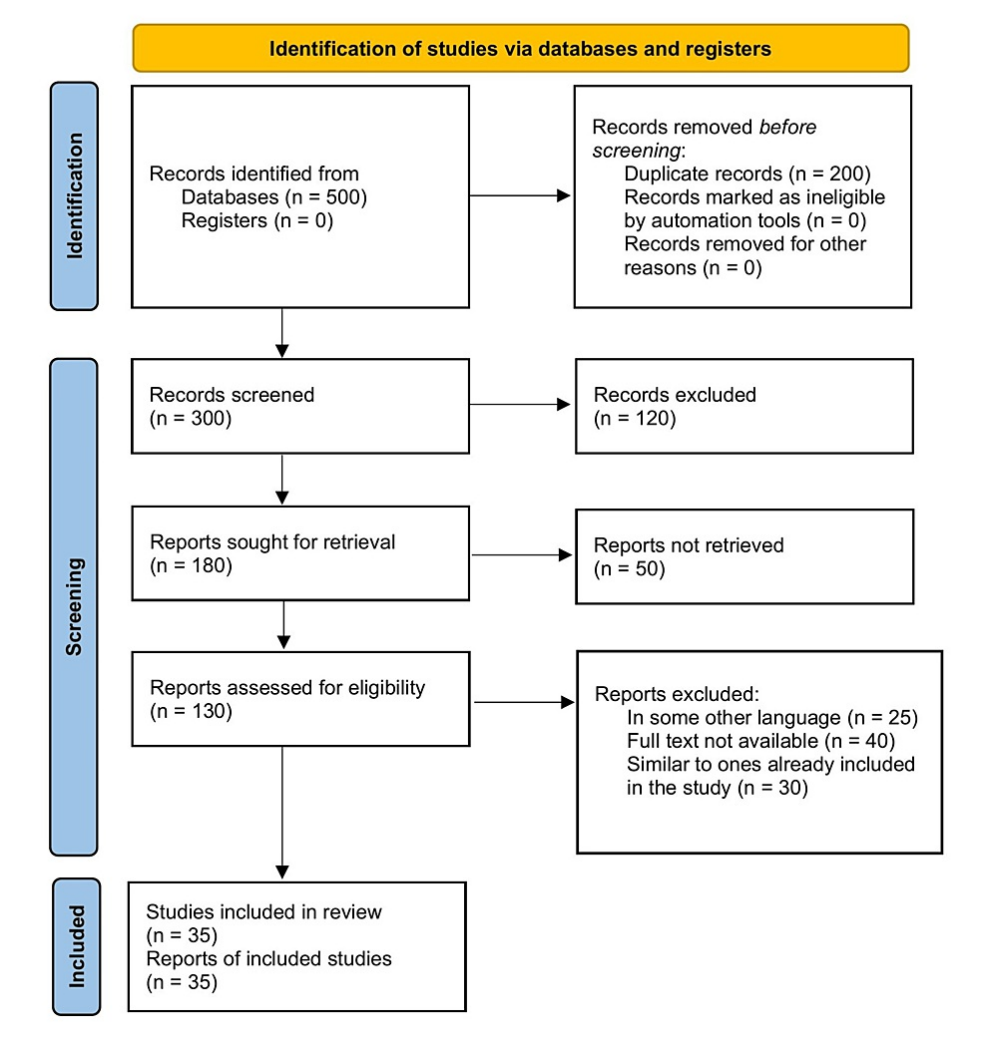
A PRISMA flow diagram for literature search PRISMA: Preferred Reporting Items for Systematic Reviews and Meta-Analyses The image has been created by the authors

A literature search was conducted to comprehensively review the effects of antibodies in serum after COVID-19 vaccine administration and their hematological and cardiovascular complications. Multiple electronic databases, including PubMed, Medical Literature Analysis and Retrieval System Online (MEDLINE), Excerpta Medica database (Embase), and Google Scholar, were searched using the following keywords and combinations: "antibodies", "immunoglobulin", "vaccine", "COVID-19", and "myocarditis". The search encompassed articles published from 2020 to 2022, and a few articles regarding the basic description of the topic were picked up from 2016 to 2022. In addition to electronic database searches, reference lists of relevant articles and review papers were manually screened to identify additional studies. The inclusion criteria involved selecting observational studies, experimental studies, systematic reviews, and meta-analyses that enlisted the various effects of antibodies in serum after COVID-19 vaccine administration and their complications. Studies with human participants were included while focusing solely on antibody effects in serum after COVID-19 vaccine administration. Only peer-reviewed, published articles were considered for inclusion. Two independent reviewers assessed the titles, abstracts, and full-text articles for eligibility, with any discrepancies resolved through discussion and consensus. The comprehensive literature search aimed to ensure the inclusion of relevant studies and provide a thorough analysis of the effects of antibodies in serum after COVID-19 vaccine administration and their hematological and cardiovascular complications. Figure 1 describes the selection process of articles used in our study.

Vaccine development and its types

Many vaccines that produce immunological reactions against the severe acute respiratory syndrome coronavirus (SARS-CoV-2) spike antigen are being developed to defend against COVID-19. The testing phase of policy formulation entails basic benchmark research and computer modeling to discover natural or synthetic antigens that can be employed as vaccine candidates, which aids in disease prevention and treatment. Pre-clinical investigations, such as systems involving the culture of cells and tissues and animal model testing, are conducted in the second phase to assess the vaccine's protection or safety as well as its capacity to elicit an immune response. We can proceed on to human clinical trials if animal safety, immunity, and success are demonstrated in small and large groups alike.

Ineffective vaccines are produced in cell culture by increasing the SARS-CoV-2 vaccine commonly in Vero cells, which are a list of cells used in cell culture, found in the kidneys of African monkeys, followed by chemical inhibition of the virus. Reduced vaccines are created by creating a virus that is less capable of reproducing itself, causing an immune response similar to that of an infection that occurs naturally but not one that results in disease. The three types of protein-containing vaccines currently in use include those based on spike proteins, receptor-binding domains, and viral-like particles (VLP). These recombined proteins are often expressed in many speech systems, including insect cells, mammal cells, plants, and yeast. Deoxyribonucleic acid (DNA) vaccines are based on the use of plasmid DNA that can be produced to a large extent within viruses. Plasmids contain the promoters of mammals and a genetic code that encapsulates spike protein; the gene is then expressed within the vaccinated person during childbirth. Ribonucleic acid (RNA) vaccines have recently been developed. They are comparable to DNA vaccines in that they provide the antigen's genetic information rather than the antigen itself, and then it is produced in the vaccinated person's nucleus. It is also possible to employ mRNA (mutation) or repeat [5, 6].

Early antibody reactions directed at SARS-CoV-2 spike protein (S) or nucleocapsid protein (NP) were found to be immunoglobulin M (IgM), IgG, and IgA isotypes with varied development rates and were discovered soon after infection that occurred naturally, around three weeks after the beginning of the symptoms. A premature antibody reaction is indicated by IgA. Antibodies having the capacity to weaken SARS-CoV-2 and prevent infection are aimed at the treatment and development of vaccines. In early January 2020, the virus's genetic sequence was established, and vaccine development began. In March 2020, the first SARS-CoV-2 vaccination clinical trials began (NCT04283461). The tests were created in such a way that the clinical phases were interspersed and the trial's execution was disrupted, with phase I/II testing proceeding by whirlwind progression into trials of phase III. By the end of the year 2020, around October 19, 212 SARS-CoV-2 vaccines had come into existence around the world; 50 of them had been clinically tested, and 162 had been under clinical development. Fourteen vaccines were inert, four were decreased, 72 were protein vaccines, 17 were DNA vaccines, 27 vaccines were based on RNA, 16 were viral-like particle-based vaccines, 26 were recurrent viral vector vaccines, and 18 were recurring viral vector vaccines. Identifying and defining effective vaccines for human use was important in prioritizing COVID-19 vaccination programs. In order to gather information and practical evidence for understanding long-term immunity through ongoing booster research studies and to anticipate the need for a revised vaccine in the wake of newly emerging versions that may evade the defence granted by the new vaccines, vaccine trials are currently ongoing in numerous locations all over the world [7-9].

Antibodies against COVID-19 

Antibody responses to SARS-CoV-2 infections are still being studied, and several studies demonstrate that people who recover generate antibodies to combat the virus. However, different groups of people show different levels of SARS-CoV-2-specific antibodies (nabs). Plasma cells and memory B cells respond to primary infection and are involved in long-term protection from re-infection. IgG antibody titers increased in the first three weeks of a study looking into antibodies from the COVID-19 infection. A study looking into COVID-19 infection antibodies found that in the initial 21 days after the appearance of symptoms, the IgG antibody levels increased, then decreased in the second month after symptoms appeared, but the level was still visible in serum and still above the IgM limit since the first-line antibody was continuously detected before IgG; it reached a peak two to five weeks later and dropped within three to five weeks after the beginning of the symptoms, depending on the group of patients. Following the onset of the disease, antibodies were found between seven and 15 days; their levels continued to rise for up to 14-22 days before measurement and began to decline. Titers in asymptomatic or clinically mild patients were low. The SARS-CoV-2 IgA-specific antibody was significantly higher in a difficult group of patients than in the weaker group [10-14]. 

Effects of vaccination 

Many vaccines have been developed that help us protect ourselves from COVID-19 by showcasing immune responses to the SARS-CoV-2 spike antigen. This can be seen by reviewing the following cases.

Study in the UK

The United Kingdom (UK) inaugurated its national vaccination program on December 8, 2020, with the approval of the Oxford-AstraZeneca ChAdOx1 nCoV-19 vaccine, which was preceded by the clearance of the Pfizer-BioNTech BNT162b2 vaccine. Initially, the vaccinations were given to key groups: home-based residents >80 years of age, healthcare workers and people at risk of illness ≥16 years, and the rest of the elderly ≥18 years. In the year 2021, in the month of April, about 31.7 million individuals (60.2% of those aged 18 and over) received the first dose of the vaccination, out of which 5.7 million individuals (10.8%) received another dose [15]. The findings revealed that seroconversion levels and multiple levels of antibody after just one vaccination were decreased in those for whom there have been no cases of infection with SARS-CoV-2 before, especially for adults >60 years. The reactions of those who received both rounds of the vaccination were higher across all age categories. Antibody levels in the body increased somewhat and then decreased following a dose of ChAdOx1 vaccination in comparison to a dose of BNT162b2, but decreased in adults after a single dose of BNT162b2. In another study comprising 3610 healthcare workers, it was discovered that the majority of persons, 99.5% and 97.1%, respectively, were converted after the first dosage of BNT162b2 or ChAdOx1, and that infected people had greater levels of IgG previously. In previously seropositive individuals, a single dosage of BNT162b2 resulted in greater antibody levels, which were equal to those of non-seronegative individuals after two doses of vaccination, as found by a multicenter study. Positive anti-spike effects of IgG increased by two to four weeks after the primary immunization compared to anti-spike IgG and varied considerably by age for participants with no indication of previous contamination. Overall, 28,144 people received an anti-spike IgG shot two to eight weeks following their initial ChAdOx1 or BNT162b2 immunization, with 24,977 (88.7%) showing absolutely zero signs concerning the previous disease and being involved in a second logistic analysis. Antibody positivity in research organizations is multivariate. A total of 20,505 participants (82.1%) demonstrated a positive anti-spike IgG response after vaccination. Seropositivity following vaccination was linked to age, gender, vaccine type, race, social stress, healthcare roles, and long-term health issues. Anti-spike IgG positivity declined as a result of the decrease in anti-spike IgG positivity, so the organization was not targeted, and seropositivity decreased rapidly in people aged > 75 years [16]. Tables 1-2 summarize the key points of the study and compare the two vaccines in terms of their responses, respectively.

**Table 1:** Key points of the study in the UK IgG: immunoglobulin G Source material: [15, 16]

Aspect	Details
UK vaccination program	Started Dec 8, 2020, with Oxford-AstraZeneca and Pfizer-BioNTech vaccines
Target groups	Initially key groups, then broader age categories
Vaccination statistics	April 2021: 31.7M received the first dose, 5.7M received the second dose
Antibody levels	Seroconversion decreased after one vaccination, higher after both
Vaccine comparison	ChAdOx1 vs. BNT162b2: varying effects on antibody levels
Health worker study	99.5% - 97.1% converted after the first dose, higher IgG in infected
Age and IgG response	Positive anti-spike IgG increased by two to four weeks, age-related variation
Total participants	28,144 received anti-spike IgG shot, 82.1% positive response
Factors affecting response	Age, gender, vaccine type, race, etc.
Decline in positivity	Positivity declined with age

**Table 2:** Comparing the Pfizer-BioNTech and Oxford-AstraZeneca vaccines in terms of their responses in different groups IgG: immunoglobulin G Source material: [15,16]

Aspect	Pfizer-BioNTech vaccine	Oxford-AstraZeneca vaccine
Approval date	Cleared after Pfizer-BioNTech	Approved Dec 8, 2020
Target groups	Administered to key groups initially	Administered to key groups initially
Vaccination statistics	31.7M received the first dose by April 2021	
Seroconversion rate	Decreased after one vaccination	Decreased after one vaccination
Antibody levels	Increased after both doses	Increased after both doses
Age impact	Positive response across age categories	Age-related variation in response
Health worker study	Higher reactions across all age groups	Similar conversion rates in health workers
IgG response	Positive anti-spike IgG after two to four weeks	Positive anti-spike IgG after two to four weeks
Total participants	28,144 received anti-spike IgG shot	
Factors affecting response	Age, gender, vaccine type, etc.	Age, gender, vaccine type, etc.
Positivity decline	No indication of decline by age	Positivity declined with age

In another study, anti-spike IgG responses to healthcare workers after either the first or both doses of the Pfizer-BioNTech or Oxford-AstraZeneca vaccine were investigated. Within 14 days of the initial immunization, IgG antibody levels increased, and from the 15th day onwards, approximately 100% of the seroconversion mutation was identified, regardless of whether the vaccine or the previous state of infection was recognized. Multiple antibody levels were reported before vaccination in 67 healthcare workers who had previously been infected and 169 who had never been affected. The prevalence of vaccination doses increased within three weeks after the initial vaccination before the onset. Those with pre-existing infections had very high titers. In the median interquartile range (IQR) anti-spike IgG studied less than three weeks after the initial vaccination, those receiving the Pfizer-BioNTech vaccine had an average infection level of 14,604 (7644-22,291) AU/mL, compared to 1028 (564-1985) AU/ml without any prior infection. Those who received the AstraZeneca vaccine showed a significantly lower incidence than those who received the Pfizer-BioNTech vaccine, with the exception of 435 (203-962) AU/mL and 10,095 (5354-17,096) AU/mL. After the Pfizer-BioNTech vaccine, significant rates were reported in small groups of previously uninfected healthcare workers. Antibodies were produced and increased in previously uninfected individuals in healthcare workers who got the second dose of the Pfizer-BioNTech vaccine, and high amounts of antibodies were detected in a small number of healthcare workers; however, there was a decline in responses between 20 and 60 days after receiving the vaccine [17]. Table 3 summarizes the same.

**Table 3:** Information about the Pfizer-BioNTech and AstraZeneca vaccines, along with their anti-spike IgG levels IgG: immunoglobulin G Source material: [17]

Vaccine type	Duration	IgG antibody levels (AU/mL)	Notes
Pfizer-BioNTech	Within 14 days	Increased IgG levels observed	
	Day 15 onwards	About 100% seroconversion rate	Irrespective of prior infection or vaccination status
	<3 weeks post-vaccination	Average level: 14,604 (7644-22,291)	Previously uninfected healthcare workers
	20-60 days post-vaccination	Decline in IgG responses	
AstraZeneca	<3 weeks post-vaccination	Average level: 435 (203-962)	Previously uninfected healthcare workers
	<3 weeks post-vaccination	Average level: 10,095 (5354-17,096)	Previously infected healthcare workers
	>60 days post-vaccination	Lower IgG levels than Pfizer	Except for the <3 weeks post-vaccination period
Pfizer-BioNTech second dose	After the second dose	Increased antibodies detected	Especially in previously

Response to CoronaVac by the Turkish Population

A chemiluminescent microparticle immunoassay was used to detect and count SARS-CoV-2 anti-spike antibodies (SARS-CoV-2 IgG II Quant; Abbott). A total of 1072 HCWs signed a consent form and answered a questionnaire at the start of the lesson. Anti-spike IgG antibodies were found in 834 of 1,072 healthcare workers after around a month of being administered CoronaVac for the first time. Seropositivity was higher among women (467 out of 552; 84.6%) than men (367 out of 520; 70.6%; p <0.001), and between the ages of 18 and 34, it was extremely high in both males and females, 88.9% and 79.5%, respectively. Anti-spike IgG antibodies were found in 75.3% of women and 64.2% of men among healthcare workers aged between 35 and 59, and 37.5% of both sexes among those aged 60 and up. In terms of antibody positivity, it turned out to be quite similar between groups of both sexes, and there seemed to be no statistical difference (p=0.05). Antibody titers were discovered to be three to four times higher in COVID-19 carriers than in non-carriers, and the difference became of statistical importance after the initial vaccination. The age range where the antibody titer was highest was 18 to 34 years. After receiving another injection of CoronaVac, 1,008 out of 1,012 healthcare workers (99.6%) developed anti-spike IgG antibodies; only four out of 1,012 (0.4%) healthcare workers continued to be seronegative [18]. Table 4 compiles the results of the study conducted in Turkey.

**Table 4:** Summary of the CoronaVac study conducted among healthcare workers in Turkey IgG: immunoglobulin G Source material: [18]

Aspect	Details
Study participants	1,072 healthcare workers in Turkey
Detection method	Chemiluminescent immunoassay (SARS-CoV-2 IgG II Quant; Abbott)
Anti-spike IgG response	834 out of 1,072 healthcare workers showed anti-spike IgG antibodies after ~one month of CoronaVac
Seropositivity by gender	Women: 84.6%, 467 out of 552; Men: 70.6%, 367 out of 520
Seropositivity by age	18-34 years: high in both males (88.9%) and females (79.5%)
Seropositivity (35-59 years)	Women: 75.3%; Men: 64.2%
Seropositivity (60+ years)	37.5% for both genders
Antibody titers	Three to four times higher in COVID-19 carriers post-vaccination, more significant after the first dose
Age with the highest titers	18-34 years
Second dose response	99.6% (1,008 out of 1,012 healthcare workers) developed anti-spike IgG antibodies after the second dose
Remaining seronegative	Only 0.4% (Four out of 1,012 healthcare workers) [14]

Studies in the United States of America

The response of the antibody to the BNT162b2 SARS-CoV-2 vaccination was measured for seronegative as well as seropositive health workers before and after two doses. Multiplex tests based on beads for measuring antibody levels in SARS-CoV-2 spike subunit proteins (spike subunit 1 (S1), spike subunit 2 (S2), receptor-binding domain (RBD)) and NP antigens were used. The same compounds were based on technology developed by Luminex xMAP and used reagent kits with non-primary antibodies, these antibodies being particular to isotypes of the immunoglobulins (IgG, IgM, and IgA). Prior to dispensing the BNT162b2 vaccine to those who had no known medical conditions or laboratory-confirmed COVID virus, peripheral blood was drawn for one to two months before the first vaccination (week 0), after the first vaccination (third week), and the second booster vaccine (sevent week) in people who did not have a known history of infection or laboratory-confirmed COVID virus used at weeks 0 and three. The researchers started by assessing immunoglobulin G antibody levels for several proteins, such as SARS-CoV-2 spike subunit proteins (S1, S2, RBD) and nucleocapsid protein (NP), three times (weeks 0, three, and seven). This was done with the help of multiplexed beads based on test binding. By week 0, the serologically negative group had the lowest median fluorescence intensity (MFI) of all four antigens (S1, 16.8; S2, 886.7; RBD, 218.2; NP, 482.9), whereas the serologically positive group had high binding antibody levels (S1, 16.8; S2, 886.7; RBD, 218.2; NP, 482.9); (S1, 10793.13; S23.12; RBD, 16409.7; NP, 20766). There is a recurrence of pre-existing antibodies when the SARS-CoV-2 infection is not present. Immunoglobulin M and IgA isotype dynamics and contributions are not well understood. Responses to S1, S2, and RBD of the immunoglobin M antibody are quite strong in seronegative persons after immunization; however, antibody levels in seropositive individuals do not increase at all after immunization and even decline by week seven. However, in people with seropositive levels, IgM initially remained significantly higher than levels in seronegative individuals exposed to two doses of vaccine (week seven). Similarly, in those who were not seronegative, the levels of IgA antibodies only rose with immunosuppression, but seropositively immunized people had no change. After the second dosage, seronegative individuals' IgM S1 antibody levels increased significantly, showing that the second vaccine had a limited or minor impact on IgM and IgA antibody responses. Vaccination did not affect those with serologically negative or positive IgM or IgA titers against NP, as expected [9]. Tables 5-6 summarize the USA study and highlight antibody levels, respectively.

**Table 5:** Summary of the study in the USA, highlighting the antibody levels after vaccination IgG: immunoglobulin G; IgA: immunoglobulin A; IgM: immunoglobulin M; S1: spike subunit 1; S2: spike subunit 2; RBD: receptor binding domain; NP: nucleocapsid protein Source material: [9]

Aspect	Details
Study focus	Antibody response to BNT162b2 SARS-CoV-2 vaccination in seronegative and seropositive health workers before and after two doses
Test method	Multiplex test using beads to measure antibody levels (IgG, IgM, and IgA) against various proteins (S1, S2, RBD, and NP)
Sample collection	Peripheral blood collected at multiple time points: week 0, week 3 (first dose), week 7 (second booster dose)
Serologically negative	Low median fluorescence intensity (MFI) for antigens at week 0; recurrence of pre-existing antibodies when SARS-CoV-2 infection absent
Serologically positive	High binding antibody levels at week 0; significant IgM levels in seropositive individuals
IgM and IgA dynamics	Not well understood; limited impact on IgM and IgA antibody responses in seronegative individuals after the second dose
Antibody levels	Strong response to S1, S2, and RBD in seronegative after immunization; no increase in seropositive levels after immunization, decline by week 7
IgM levels (week 7)	Significantly higher in seropositive compared to seronegative; the limited impact of the second dose on IgM responses
IgA levels (week 7)	Rise only with immunosuppression in non-seronegative; no change in seropositive immunized individuals
IgM/IgA against NP	No significant effect of vaccination on serologically negative/positive IgM or IgA titers against NP

**Table 6:** Antibody levels at different weeks IgG: immunoglobulin G; IgA: immunoglobulin A; IgM: immunoglobulin M; S1: spike subunit 1; S2: spike subunit 2; RBD: receptor binding domain; NP: nucleocapsid protein Source material: [9]

Antibody levels	Seronegative at week 0	Seropositive at week 0	Seronegative after two doses (week 7)	Seropositive after two doses (week 7)
S1 (IgG)	Low	High	Strong response	No increase, decline by week 7
S2 (IgG)	Low	High	Strong response	No increase, decline by week 7
RBD (IgG)	Low	High	Strong response	No increase, decline by week 7
NP (IgG)	Low	High	Strong response	No increase, decline by week 7
IgM (S1)	N/A	Significant	Limited impact on response	Significantly higher than seronegative
IgA (S1)	N/A	N/A	Rise only with immunosuppression	No change in seropositivity

Complications of the vaccine 

The global crisis accelerated the process of vaccine development and use, so innovative vaccines based on mRNA continue to be the most profitable method of averting the propagation of COVID-19 infection while it still continues to be active. Although COVID-19 vaccines offer a reduced burden of illness, if not full immunity, they may also be associated with their own negative effects. The scientific literature has detailed a number of unfavorable events that happened during this process, leading potential vaccine recipients to doubt the ostensible safety statistics [19-21]. Current surveillance studies in a mass immunization setting are currently researching occasional problems such as inflammation of the myocardium and pericardium and thromboembolic events connected concerning these vaccines after administration of the mRNA-based COVID-19 vaccines BNT162b2 (Pfizer-BioNTech) and mRNA-1273 (Moderna) [22]. According to a different study, some people may experience an uncommon incidence of immune thrombotic thrombocytopenia after receiving the ChAdOx1 nCov-19 vaccination. This condition is brought on by platelet-activating antibodies against platelet factor -4 (PF4) and has clinical similarities to autoimmune heparin-induced thrombocytopenia [23].

Etiology, Clinical Features and Management of COVID-19 Vaccine-Related Myocarditis

Younger men under 30 seem to be more susceptible to post-vaccination myocarditis, and its incidence seems to occur more frequently in individuals who have received the second injection of the COVID-19 mRNA vaccines, usually within less than a week of vaccination. The COVID-19 mRNA vaccines have caused myocarditis, but the exact cause is still unknown. It may be related to the vaccine's active ingredient, which is a SARS-CoV-2 spike glycoprotein-coding nucleoside-modified mRNA, the immunological response that results after immunization, or the dysregulated generation of cytokines [24-27]. The initial diagnosis of either COVID-19 or its vaccine-induced myocardial inflammation is made using clinical suspicion, analysis done in labs, cardiac biomarkers, and imaging techniques like cardiac echocardiography, fluorodeoxyglucose positron emission tomography (PET), or cardiovascular magnetic resonance (CMR), of which CMR is the minimally invasive investigation of superior quality (the gold standard) for determining the presence of inflammatory cardiomyopathies. Rarely, confirming the diagnosis may involve an endomyocardial biopsy, especially if giant cell myocarditis is being examined [28]. Most patients elicit symptoms like palpitations and acute chest pain, and on doing an electrocardiogram workup, abnormal findings and evidence of myocardial injury and damage as shown by elevated levels of the troponin enzymes have been observed; however, there is no evidence of stenosis, which may limit the flow of blood or any culprit lesion on coronary angiography. In extreme situations, endomyocardial biopsy should be taken into consideration so that the diagnosis can be confirmed and direct additional treatment can be started [29,30]. Inflammation of the myocardium and the pericardium typically has a good short-term prognosis, is moderate, subsides on its own, and responds favorably to a wide range of therapies that do not involve surgery, like proper rest, colchicine, nonsteroidal anti-inflammatory medications, and other supportive measures. Cardioprotective drugs could be taken into consideration for individuals who have left ventricular systolic dysfunction, although prospective or randomized studies are sparse in this area. It is possible to investigate methylprednisolone therapy, which is a corticosteroid with high doses for severe inflammation of the myocardium brought on by COVID-19 vaccines, particularly when there is a lack of viral genetic material in the tissue. Various case reports show that intravenous immunoglobulins were only sometimes utilized to treat myocarditis following COVID-19 immunization, apart from the cardioprotective medications and the supportive care given. Lastly, according to professional opinion, refraining from or postponing competitive sports and high-intensity exercise until full recovery, defined as a decline in symptomatology and the signs, having stable vitals, and normal values of the cardiac biomarkers and diagnostic abnormalities, has occurred [20,27]. 

Etiology, Clinical Features, and Management of COVID-19 Vaccine-Related Thrombotic Events

Approximately one to two weeks after receiving the SARS-CoV-2 vaccine with ChAdOx1nCov-19, a clinical spectrum of thrombocytopenia ranging from moderate to severe densities of decline in the thrombocytes and thrombotic difficulties at unusual locations begin,s and indicate a condition that, on a clinical basis, mimics an extremely serious disease called heparin-induced thrombocytopenia, an established prothrombotic disease that occurs due to immunoglobulins that activate platelets that detect multimolecular aggregates between negative PF4 [31]. Clinicians should be mindful of the fact that venous or arterial thrombosis, which manifests clinically five to 20 days following vaccination, can occur in certain patients at uncommon places such as the brain or belly. An unfavorable response to the prior vaccine given for COVID-19 may be observed when it is accompanied by thrombocytopenia. It is possible to check patients for a potential post-vaccination decrease in platelet count or thrombosis using an enzyme-linked immunosorbent assay (ELISA) or by using a PF4-enhanced platelet activation assay to detect antibodies against PF4. Even though intravenous immunoglobulin administration and the initiation of anticoagulation do not require laboratory diagnosis, the identification of cases and risk-to-benefit analyses in the future of this and other vaccines will depend heavily on the detection of these unusual platelet-activating antibodies. Intravenous immunoglobulin in higher doses has caused hypercoagulability to decelerate and thrombocyte counts to rise quickly [32,33,23]. In patients, the outcomes of intravenous immunoglobulin treatment were as follows: excellent: when the platelet count increased by at least 100×10^9^/L within the subsequent week; good: if the rise was between 50 and 99×10^9^/L; and poor: when the platelet count response was less than 50×10^9^/L. For individuals with severe illnesses, it is suggested that a a thorough prospective study on the use of intravenous immunoglobulin in individuals unresponsive to conventional therapy is warranted. Individual risk-benefit analyses should be used to make treatment decisions because intravenous immunoglobulin therapy alone may predispose to thromboembolism [34-39]. 

## Conclusions

The deadly COVID-19 pandemic is caused by the SARS-CoV-2 virus. Over 6.86 million people have died due to the deadly infection, and 660 million people globally have been affected. The virus is spread mainly through respiratory drops and fomites. The most common preventative measures are hand washing, applying a face mask, and keeping away from the public.

Antibodies can weaken SARS-CoV-2 and prevent infection, which can help with treatment and vaccine development. In early January 2020, the virus's genetic sequence was established, and vaccine development began. Fifty of the 212 SARS-CoV-2 vaccines being created all over the globe were clinically evaluated as of October 19, 2020, and 162 had been created prior to treatment. Special SARS-CoV-2 antibodies are found in varying amounts in different groups of people (nabs). Plasma cells and memory B cells are crucial in long-term defense against re-infection and respond to primary infection. Vaccines provide protection against the coronavirus by eliciting an immunological response against the anti-spike antigen of the virus. Ineffective vaccines were produced by cell culture by increasing the SARS-CoV-2 vaccine normally in Vero cells. Reduced vaccinations are made by producing a virus with a weakened variant that can only replicate itself to a limited extent and, as a result, does not cause disease or infection but causes an allergic reaction that is similar to that caused by any natural infection. Spike-protein-based vaccines, RBD-based vaccines, and virus-like particle-based (VLP) vaccinations form the triad of the most common forms of recombinant protein vaccines available. Immunoglobulin M is the first antibody to be released in humans after vaccination because it is the first line of defense, according to the results of a sample study. However, IgG titers are not the highest in number, especially in the age groups of 18 to 36 years. As people get older, the number of titers declines, with a nearly nonexistent response in adults over the age of 76.

Some instances of complications have been reported after receiving the vaccination shot, which include various cardiovascular and hematological complications like myocarditis and vaccine-induced thrombotic thrombocytopenia; however, they are also largely treatable using proper medication and medical attention, ensuring the recovery of the individual who suffered them, as we see in any other vaccine. However, the effects of the vaccine supercede its adverse effects, and hence it should be continued to be taken by all individuals in effect for their safety and benefit and to establish herd immunity to protect themselves and the world from the catastrophic virus.
